# Network pharmacology of iridoid glycosides from *Eucommia ulmoides* Oliver against osteoporosis

**DOI:** 10.1038/s41598-022-10769-w

**Published:** 2022-05-06

**Authors:** Ting Wang, Liming Fan, Shuai Feng, Xinli Ding, Xinxin An, Jiahuan Chen, Minjuan Wang, Xifeng Zhai, Yang Li

**Affiliations:** 1 Biomedicine Key Laboratory of Shaanxi Province, College of Life Sciences, Northwest University, Xi’an, 710069 China; 2 Physical and Chemical Laboratory, Shaanxi Provincial Center for Disease Control and Prevention, Xi’an, 710054 China; 3 School of Pharmaceutical Sciences, Xi’an Medical University, Xi’an, 710021 China

**Keywords:** Computational biology and bioinformatics, Drug discovery, Endocrinology

## Abstract

*Eucommia ulmoides* Oliver is one of the commonly used traditional Chinese medicines for the treatment of osteoporosis, and iridoid glycosides are considered to be its active ingredients against osteoporosis. This study aims to clarify the chemical components and molecular mechanism of iridoid glycosides of *Eucommia ulmoides* Oliver in the treatment of osteoporosis by integrating network pharmacology and molecular simulations. The active iridoid glycosides and their potential targets were retrieved from text mining as well as Swiss Target Prediction, TargetNet database, and STITCH databases. At the same time, DisGeNET, GeneCards, and Therapeutic Target Database were used to search for the targets associated with osteoporosis. A protein–protein interaction network was built to analyze the interactions between targets. Then, DAVID bioinformatics resources and R 3.6.3 project were used to carry out Gene Ontology enrichment analysis and Kyoto Encyclopedia of Genes and Genomes pathway analysis. Moreover, interactions between active compounds and potential targets were investigated through molecular docking, molecular dynamic simulation, and binding free energy analysis. The results showed that a total of 12 iridoid glycosides were identified as the active iridoid glycosides of *Eucommia ulmoides* Oliver in the treatment of osteoporosis. Among them, aucubin, reptoside, geniposide and ajugoside were the core compounds. The enrichment analysis suggested iridoid glycosides of *Eucommia ulmoides* Oliver prevented osteoporosis mainly through PI3K-Akt signaling pathway, MAPK signaling pathway and Estrogen signaling pathway. Molecular docking results indicated that the 12 iridoid glycosides had good binding ability with 25 hub target proteins, which played a critical role in the treatment of osteoporosis. Molecular dynamic and molecular mechanics Poisson–Boltzmann surface area results revealed these compounds showed stable binding to the active sites of the target proteins during the simulations. In conclusion, our research demonstrated that iridoid glycosides of *Eucommia ulmoides* Oliver in the treatment of osteoporosis involved a multi-component, multi-target and multi-pathway mechanism, which provided new suggestions and theoretical support for treating osteoporosis.

## Introduction

Osteoporosis (OP) is a chronic epidemic characterized by low bone mass and deterioration of bone microarchitecture, which leads to increased bone fragility and fracture risk, and caused a heavy economic burden to society^1^. The etiology of OP is very complex, including the interaction of endocrine, nutritional status, genetic, physiological and immune factors^2,3^. The imbalance between bone formation of osteoblasts and bone resorption of osteoclasts is the underlying cause of OP^4^. The treatment of OP depends on drug therapy, including bisphosphonate, selective estrogen receptor modulator, mixed steroid receptor agonist, monoclonal antibody against RANKL, parathyroid hormone analogue and so on^5^. These drugs can alleviate bone loss and improve clinical symptoms to a certain extent, but their long-term clinical application is limited by low tolerance, severe side effects and high cost^6^. Therefore, it is of great significance to develop more safe, effective and economical drugs for the treatment of OP.

Traditional Chinese medicine (TCM) has a long history in China. It is more and more popular with the advantages of good curative effect, few side effects and affordable price^7^. In the theoretical system of TCM, OP is recognized as bone atrophy or arthralgia syndrome caused by kidney deciency^8^. *Eucommia ulmoides* Oliver (EU) is one of the most important nourishing medicinal materials in TCM. It has been found that EU can effectively prevent bone loss, improve bone biomechanical strength, prevent the deterioration of trabecular bone microarchitecture and cure OP^9–11^. Modern researches have considered that iridoid glycosides are the main pharmacological ingredients of EU^12–14^. There are many studies showing that iridoid glycosides play a primary role in bone resorption and bone remodeling. Iridoid glycosides can increase the differentiation and activity of osteoblasts, promote bone formation, inhibit the generation of osteoclasts, reduce osteoclast activity and limit bone resorption^15^. Such as aucubin could improve osteoblast differentiation in MG63 cells^16^. Geniposide could induce the proliferation and differentiation of the MC3T3-E1 cells^17^. Catalpol could suppress osteoclastogenesis and attenuate osteoclast-derived bone resorption^18^. Asperuloside could inhibit osteoclasts differentiation and reduce the number of osteoclasts^19^. Monotropein could promote the formation of osteoblastic and decreases the production of pro-inflammatory cytokines in osteoblasts^20^. Swertiamarin could significantly increase the expression level of OPG and against the activity of osteoclast^21^. These results indicated that iridoid glycosides exhibited potential preventive and therapeutic effects on OP. However, there are few studies on the chemical components and molecular mechanisms of iridoid glycosides of *Eucommia ulmoides* Oliver (IGEUs) in the treatment of OP.

**Figure 1:**
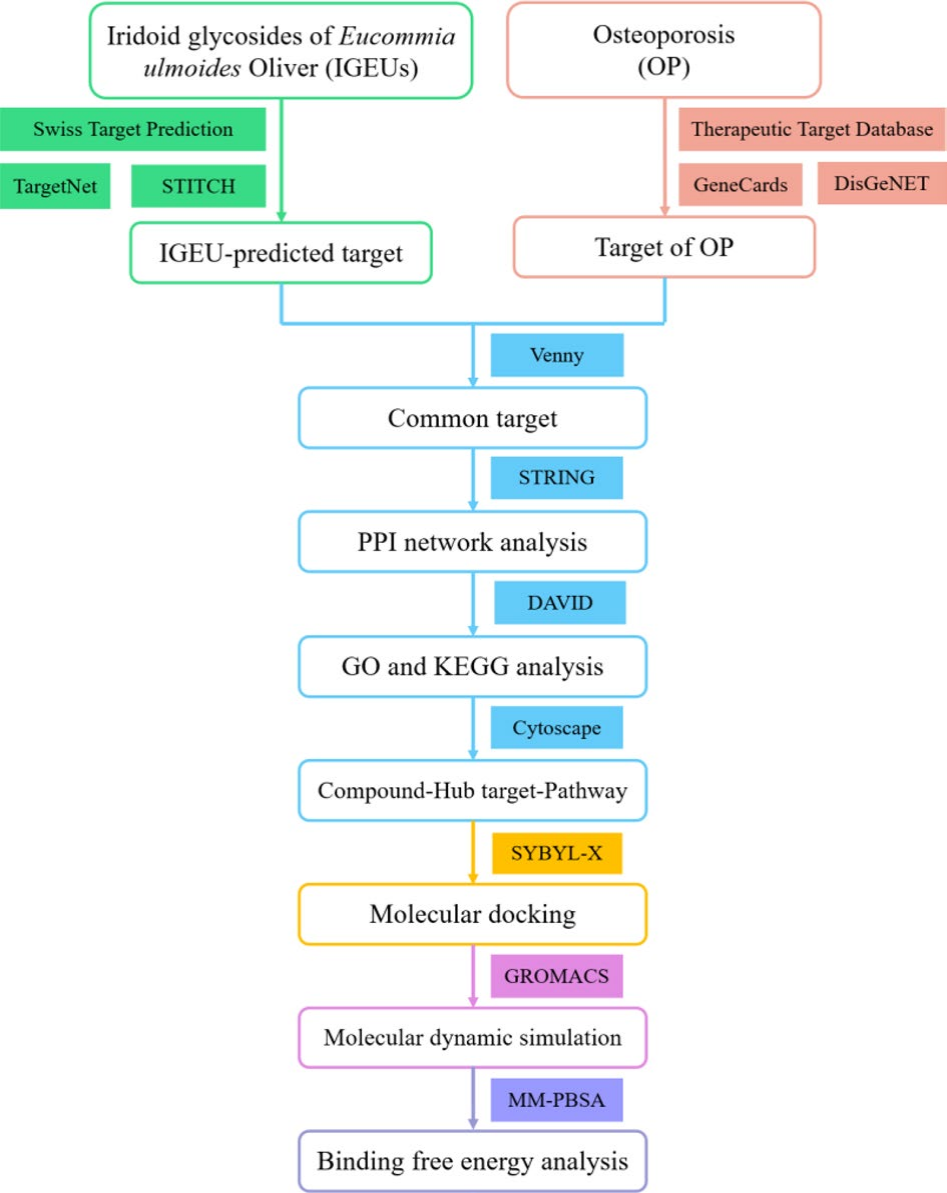
Flowchart of iridoid glycosides of *Eucommia ulmoides* Oliver in the treatment of OP.

In view of the complex chemical composition of TCM, network pharmacology has become a powerful tool to explore TCM from the system and molecular level^22,23^. Molecular docking is an important method to verify the reliability of network pharmacology in predicting drugs and targets^24^. Molecular dynamic (MD) simulation is frequently used to observe the dynamic process of complex conformations and provide more realistic trajectories over time^25^. Therefore, the purpose of this study is to clarify the hypothesis of a multi-component, multi-target and multi-pathway mechanism of IGEUs in the treatment of OP through network pharmacology, molecular docking and MD simulation, so as to provide a theoretical basis for future research. The specific research process is shown in Fig. 1.

## Materials and methods

### Collection of IGEUs components and targets

In this study, the IGEUs were obtained from China National Knowledge Infrastructure (https://www.cnki.net/), PubMed (https://pubmed.ncbi.nlm.nih.gov/) and Web of Science (https://www.webofscience.com/) databases^26,27^, which summarized the contents, biological functions and pharmacological effects of main active components from different parts of EU^28–36^. In addition, the PubChem ID, CAS, canonical smiles and 2D structure of the IGEUs were obtained from the PubChem database (https://pubchem.ncbi.nlm.nih.gov/)^37^. The targets of the IGEUs were predicted by using the Swiss Target Prediction webserver (http://www.swisstargetprediction.ch/), the TargetNet database (http://targetnet.scbdd.com/) and the STITCH webserver (http://stitch.embl.de/)^38–40^. The IGEU-predicted target network is based on the active components of IGEUs and their potential targets. The targets related to OP were obtained from the DisGeNET database (https://www.disgenet.org/), GeneCards database (https://www.genecards.org/) and Therapeutic Target Database (http://db.idrblab.net/ttd/)^41–43^. “Osteoporosis” was the keyword and “*Homo sapiens*” was the organism used when searching for the targets, which were verified using UniProt ID, and the target names were standardized into official gene symbols with the UniProt database (https://www.uniprot.org/)^44^. The common targets between the compounds and the disease were obtained by Venny2.1.0 (https://bioinfogp.cnb.csic.es/tools/venny/index.html) with a Venn diagram^45^.

### Collection of protein–protein interaction (PPI) data

The common targets between IGEUs and OP were imported to the STRING11.0 database (https://string-db.org/) to construct a protein–protein interaction (PPI) network^46^. The organism was chosen as “*Homo sapiens*”, the minimum interaction threshold was selected as “medium confidence > 0.4”, disconnected nodes in the network were hidden, and other parameters remained the default settings. Cytoscape 3.8.0 was used to construct and visualize the PPI network^47^. At the same time, we analyzed the PPI network using the plug-in of “Analyze Network” tool. Network topology analysis, which contains many topological parameters, was applied to network pharmacology, among which edgecount is the most important parameter^48^. The edgecount of a node refers to the number of other nodes that interact with it^49^. We selected the nodes with more than twice the median edgecount of all nodes to construct the hub PPI. The correlated targets in hub PPI were identified as the hub targets.

### GO and KEGG enrichment analyses

In this study, the hub targets of IGEUs in the treatment of OP were introduced into DAVID bioinformatics resources (https://david.ncifcrf.gov/home.jsp) to analyze the Gene Ontology (GO) annotation and Kyoto Encyclopedia of Genes and Genomes (KEGG) pathway (www.kegg.jp/kegg/kegg1.html)^50,51^. The adjusted *p*-value was used to save the enrichment analysis results of GO and KEGG pathway, and the threshold of statistical significance was set to *p* < 0.05. Use R 3.6.3 (https://www.r-project.org/) software to visualize the results. The compound—hub target—pathway network was constructed by Cytoscape 3.8.0 (https://cytoscape.org/). We defined the compounds with edgecount greater than twice the median edgecount as the core compounds in this network for further analysis.

### Molecular docking

Molecular docking was used to confirm the interactions between the compounds and the hub targets of IGEUs in treating OP and to verify the accuracy of the network pharmacology prediction. The 3D structures of the target proteins were downloaded from the RCSB database (https://www.rcsb.org/), and the MOL2 structures of the compounds were downloaded from the TCMSP database (https://tcmspw.com/tcmsp.ph)^52,53^. SYBYL-X 2.1.1 was used to carry out the molecular docking of core compounds (ligands) with hub targets (receptors) and determine their binding activity^54^. The Total score is a complex score obtained by docking the receptor and ligand with corresponding parameters using Surflex-Dock procedure. It is generally believed that when the conformation of the ligand and receptor complex is stable, the higher the Total score, the higher the affinity of the receptor and ligand^55^. Total Score > 4.0 indicates certain binding activity, Total Score > 5.0 indicates good binding activity, while Total Score > 7.0 indicates strong binding activity^56^. PyMOL 2.4 (https://pymol.org) software was used to visualize the docking results^57^.

### Molecular dynamic simulation and binding free energy analysis

The selected compounds were prepared with ATB webserver (http://atb.uq.edu.au/) prior to MD simulation to generate an initial topology for the ligands^58^. MD simulation of the complex was performed using GROMACS 2019.6^59^. GROMOS96 54a7 force field was applied to the system, and dodecahedron water box consisting of a TIP3P water model was used to solvate the system. Na^+^ and Cl^−^ ions were also solvated in the box to neutralize the system charge. Next, energy optimization process was performed using the steepest descent method. For equilibration simulation, 100 ps NVT equilibration was performed by using the velocity rescaling thermostat coupling method to keep the temperature constant at 300 K. Then, 100 ps NPT equilibration was performed. To treat the long-range coulombic interactions, the PME method was used. Production run of MD simulation was performed till 50 ns for each protein–ligand complex. Consequently, root mean square deviation (RMSD), root-mean-square fluctuation (RMSF), the radius of gyration (Rg), solvent-accessible surface area (SASA) and hydrogen bonds were calculated according to the trajectory for further analysis. The binding free energies (ΔG_bind_) including electrostatic interactions (ΔE_elec_), Vander Waals interactions (ΔE_vdW_), non-polar solvation energy (ΔG_SASA_) and polar solvation energy (ΔG_polar)_ were calculated using the molecular mechanics Poisson–Boltzmann surface area (MM-PBSA) method implemented in GROMACS compatible tool “g_mmpbsa”^60,61^. In addition, the energy of each residue was decomposed, and the energy decomposition could be analyzed to determine the contribution of key residues to binding.

## Results

### Collection and screening of active IGEUs and construction of networks

**Figure 2:**
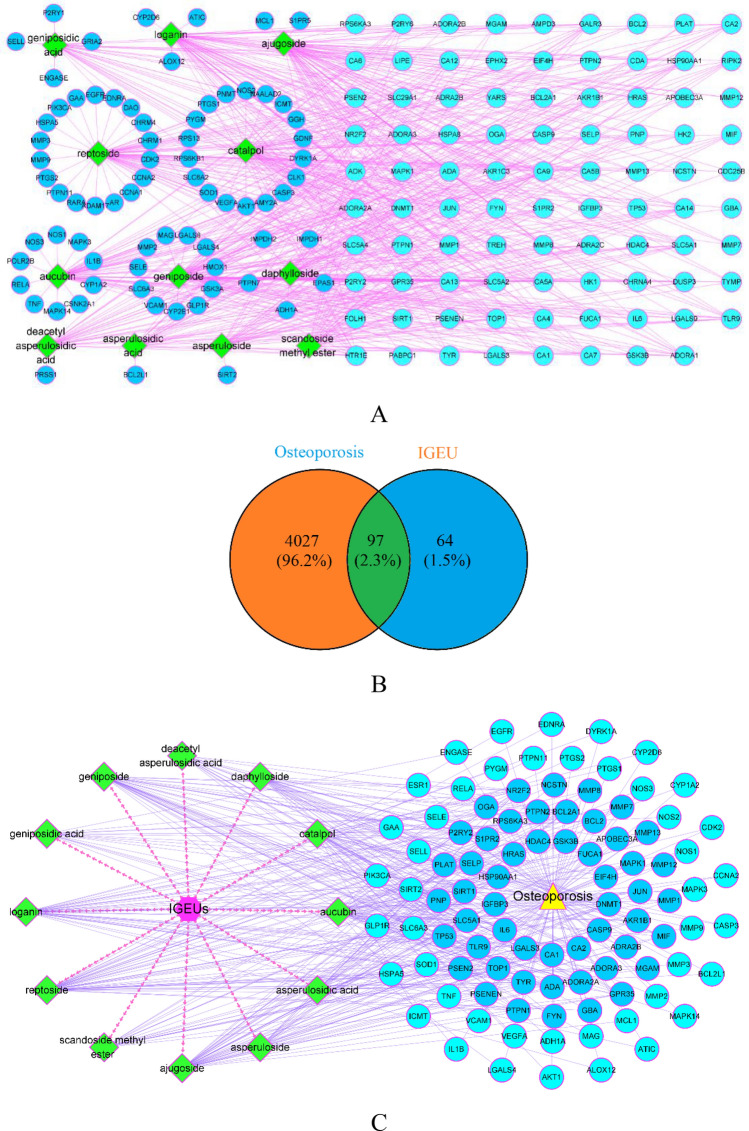
(**A**) IGEU- predicted target network. Green nodes represent the active IGEUs, cyan nodes represent shared targets, and blue nodes represent unique targets exist in only one compound. The purple edges represent the interaction between compounds and targets. (**B**) Venn analysis diagram of IGEUs with OP. Orange section represents the potential targets of OP, blue section represents the potential targets of IGEUs, and green section represents the common targets between OP and IGEUs. (**C**)The compound—target—disease network of IGEUs in the treatment of OP. Purple node represents IGEUs, green nodes represent the active compounds of IGEUs, yellow node represents osteoporosis, and blue nodes represent the anti-OP targets of the active compounds. The red virtual arrows represent the interactions between IGEUs and its compounds, and the light purple edges represent the interactions among compounds, targets and disease. [(A&C) were created from Cytoscape 3.8.0 (https://cytoscape.org), B was made in Venny2.1.0 (https://bioinfogp.cnb.csic.es/tools/venny/index.html)].

After text mining and removal of non-target compounds reported in the literatures, 12 iridoid glycosides were obtained as active IGEUs (Table 1). In total, 161 targets of IGEUs were identified. The compound–target network is composed of 175 nodes and 554 edges (Fig. 2A). By searching the disease-related database, a total of 4124 targets of OP were integrated. Finally, 97 common targets were identified as therapeutic targets for the anti-OP activity of IGEUs (Fig. 2B). Moreover, a compound–target–disease network with 111 nodes and 414 edges was constructed using IGEUs, osteoporosis and common targets (Fig. 2C).

**Table 1:** Identification of 12 iridoid glycosides from IGEUs by text mining.

NO	Compounds	CAS	Formula	Molecular Weight(g/mol)	2D Structure	References
1	Aucubin	479-98-1	C_15_H_22_O_9_	346.33		^30–34,36^
2	Catalpol	2415-24-9	C_15_H_22_O_10_	362.33		^29,30,36^
3	Ajugoside	52,916-96-8	C_17_H_26_O_10_	390.4		^30,33^
4	Asperuloside	14,259-45-1	C_18_H_22_O_11_	414.4		^30,31,34–36^
5	Asperulosidic acid	25,368-11-0	C_18_H_24_O_12_	432.4		^29–31,34,36^
6	Deacetyl asperulosidic acid	14,259-55-3	C_16_H_22_O_11_	390.34		^31,34–36^
7	Geniposide	24,512-63-8	C_17_H_24_O_10_	388.4		^29,30,35,36^
8	Geniposidic acid	27,741-01-1	C_16_H_22_O_10_	374.34		^28–32,34–36^
9	Reptoside	53,839-03-5	C_17_H_26_O_10_	390.4		^29,33,36^
10	Daphylloside	14,260-99-2	C_19_H_26_O_12_	446.4		^30,35,36^
11	Scandoside methyl ester	27,530-67-2	C_17_H_24_O_11_	404.4		^30,35^
12	Loganin	18,524-94-2	C_17_H_26_O_10_	390.4		^30,35,36^

### PPI network of the anti-OP targets of IGEUs

**Figure 3:**
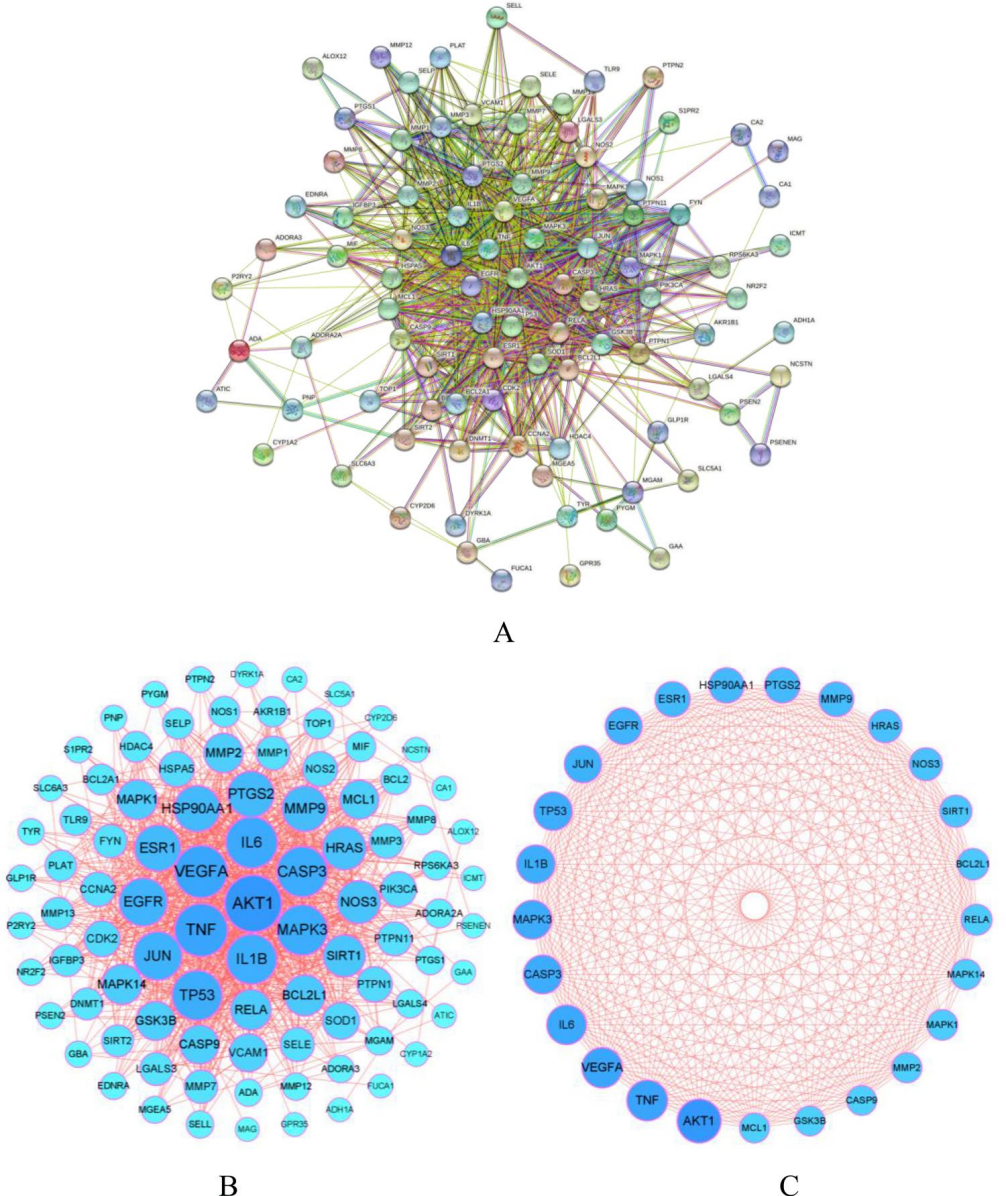
(**A**) Original PPI network from the database. (**B**) The optimized PPI network. The sizes and colors of the nodes were proportional to the edgecounts. The larger the node, the darker the color, and the stronger the interaction, indicating that the interaction played a more central role in the PPI network. (**C**) The hub PPI network. The sizes and colors were proportional to the edgecounts. [A was obtained from STRING11.0 database (https://string-db.org/), (**B**,**C**) were obtained from Cytoscape 3.8.0 (https://cytoscape.org)].

The 97 common targets were imported into the STRING11.0 database to construct the PPI network. After removing 4 disconnected nodes, there were 93 nodes left, but the original PPI network was usually rough (Fig. 3A). Therefore, a second PPI network was constructed by Cytoscape 3.8.0 in order to obtain a better visualization and understanding. The results showed that the reconstructed PPI network included 93 nodes and 934 edges (Fig. 3B). The edgecount of each node in the PPI network was shown in Table 2, the edgecount of the median node is 15. A total of 25 hub targets representing protein–protein interactions were used to construct the hub PPI network (Fig. 3C) and the topological parameters of the hub PPI network were shown in Table 3. The hub targets were AKT1, TNF, VEGFA, IL6, MAPK3, CASP3, IL1B, TP53, JUN, EGFR, PTGS2, HSP90AA1, ESR1, MMP9, HRAS, NOS3, SIRT1, BCL2L1, RELA, MAPK1, MAPK14, MMP2, GSK3B, CASP9 and MCL1.

**Table 2:** The edgecounts of the targets in the PPI network.

NO	Gene name	Protein name	Edgecount	NO	Gene name	Protein name	Edgecount
1	AKT1	Protein kinase	67	48	HDAC4	Histone deacetylase 4	15
2	TNF	Tumor necrosis factor	59	49	BCL2A1	Bcl-2-related protein A1	15
3	VEGFA	Vascular endothelial growth factor A	58	50	TLR9	Toll-like receptor 9	15
4	IL6	Interleukin-6	56	51	TOP1	DNA topoisomerase 1	14
5	MAPK3	Mitogen-activated protein kinase 3	54	52	AKR1B1	Aldo–keto reductase family 1 member B1	14
6	CASP3	Caspase-3	54	53	MIF	Macrophage migration inhibitory factor	13
7	IL1B	Interleukin-1 beta	53	54	BCL2	Apoptosis regulator Bcl-2	13
8	TP53	Cellular tumor antigen p53	53	55	MMP8	Neutrophil collagenase	12
9	JUN	Transcription factor AP-1	50	56	ADORA2A	Adenosine receptor A2a	11
10	EGFR	Epidermal growth factor receptor	49	57	RPS6KA3	Ribosomal protein S6 kinase alpha-3	11
11	PTGS2	Prostaglandin G/H synthase 2	45	58	LGALS4	Galectin-4	10
12	HSP90AA1	Heat shock protein HSP 90-alpha	45	59	MGAM	Maltase-glucoamylase, intestinal	10
13	ESR1	Estrogen receptor	45	60	PTGS1	Prostaglandin G/H synthase 1	10
14	MMP9	Matrix metalloproteinase-9	44	61	ADA	Adenosine deaminase	8
15	HRAS	GTPase HRas	40	62	ADORA3	Adenosine receptor A3	8
16	NOS3	Nitric oxide synthase, endothelial	39	63	SELL	L-selectin	8
17	SIRT1	NAD-dependent protein deacetylase sirtuin-1	37	64	MMP12	Macrophage metalloelastase	8
18	BCL2L1	Bcl-2-like protein 1	35	65	EDNRA	Endothelin-1 receptor	7
19	RELA	Transcription factor p65	34	66	MGEA5	Protein O-GlcNAcase	7
20	MAPK1	Mitogen-activated protein kinase 1	33	67	P2RY2	P2Y purinoceptor 2	6
21	MAPK14	Mitogen-activated protein kinase 14	33	68	TYR	Tyrosinase	6
22	MMP2	72 kDa type IV collagenase	32	69	GLP1R	Glucagon-like peptide 1 receptor	6
23	GSK3B	Glycogen synthase kinase-3 beta	31	70	NR2F2	COUP transcription factor 2	6
24	CASP9	Caspase-9	31	71	PSEN2	Presenilin-2	6
25	MCL1	Induced myeloid leukemia cell differentiation protein Mcl-1	31	72	GBA	Lysosomal acid glucosylceramidase	6
26	VCAM1	Vascular cell adhesion protein 1	29	73	PNP	Purine nucleoside phosphorylase	5
27	SOD1	Superoxide dismutase	27	74	SLC6A3	Sodium-dependent dopamine transporter	5
28	CDK2	Cyclin-dependent kinase 2	25	75	PYGM	Glycogen phosphorylase, muscle form	5
29	PTPN1	Tyrosine-protein phosphatase non-receptor type 1	24	76	PTPN2	Tyrosine-protein phosphatase non-receptor type 2	5
30	PIK3CA	PI3-kinase subunit alpha	24	77	S1PR2	Sphingosine 1-phosphate receptor 2	5
31	HSPA5	Endoplasmic reticulum chaperone BiP	24	78	DYRK1A	Dual specificity tyrosine-phosphorylation-regulated kinase 1A	4
32	NOS2	Nitric oxide synthase, inducible	23	79	CA2	Carbonic anhydrase 2	3
33	CCNA2	Cyclin-A2	22	80	CYP2D6	Cytochrome P450 2D6	3
34	MMP3	Stromelysin-1	22	81	SLC5A1	Sodium/glucose cotransporter 1	3
35	MMP1	Interstitial collagenase	21	82	NCSTN	Nicastrin	3
36	SELE	E-selectin	21	83	ATIC	Bifunctional purine biosynthesis protein ATIC	2
37	PTPN11	Tyrosine-protein phosphatase non-receptor type 11	21	84	CYP1A2	Cytochrome P450 1A2	2
38	MMP7	Matrilysin	20	85	CA1	Carbonic anhydrase 1	2
39	FYN	Tyrosine-protein kinase Fyn	20	86	ALOX12	Polyunsaturated fatty acid lipoxygenase ALOX12	2
40	LGALS3	Galectin-3	19	87	ICMT	Protein-S-isoprenylcysteine O-methyltransferase	2
41	SIRT2	NAD-dependent protein deacetylase sirtuin-2	17	88	GAA	Lysosomal alpha-glucosidase	2
42	MMP13	Collagenase 3	17	89	PSENEN	Gamma-secretase subunit PEN-2	2
43	DNMT1	DNA (cytosine-5)-methyltransferase 1	17	90	ADH1A	Alcohol dehydrogenase 1A	1
44	IGFBP3	Insulin-like growth factor-binding protein 3	17	91	FUCA1	Tissue alpha-L-fucosidase	1
45	PLAT	Tissue-type plasminogen activator	16	92	MAG	Myelin-associated glycoprotein	1
46	NOS1	Nitric oxide synthase, brain	15	93	GPR35	G-protein coupled receptor 35	1
47	SELP	P-selectin	15

**Table 3:** The topological parameters of hub PPI network.

Network parameters	Value
Number of nodes	25
Number of edges	456
Clustering coefficient	0.987
Network diameter	2
Network radius	1
Network density	0.987
Characteristic path length	1.014
Avg. number of neighbors	20.727
Connected components	1

### GO and KEGG enrichment analyses

**Figure 4:**
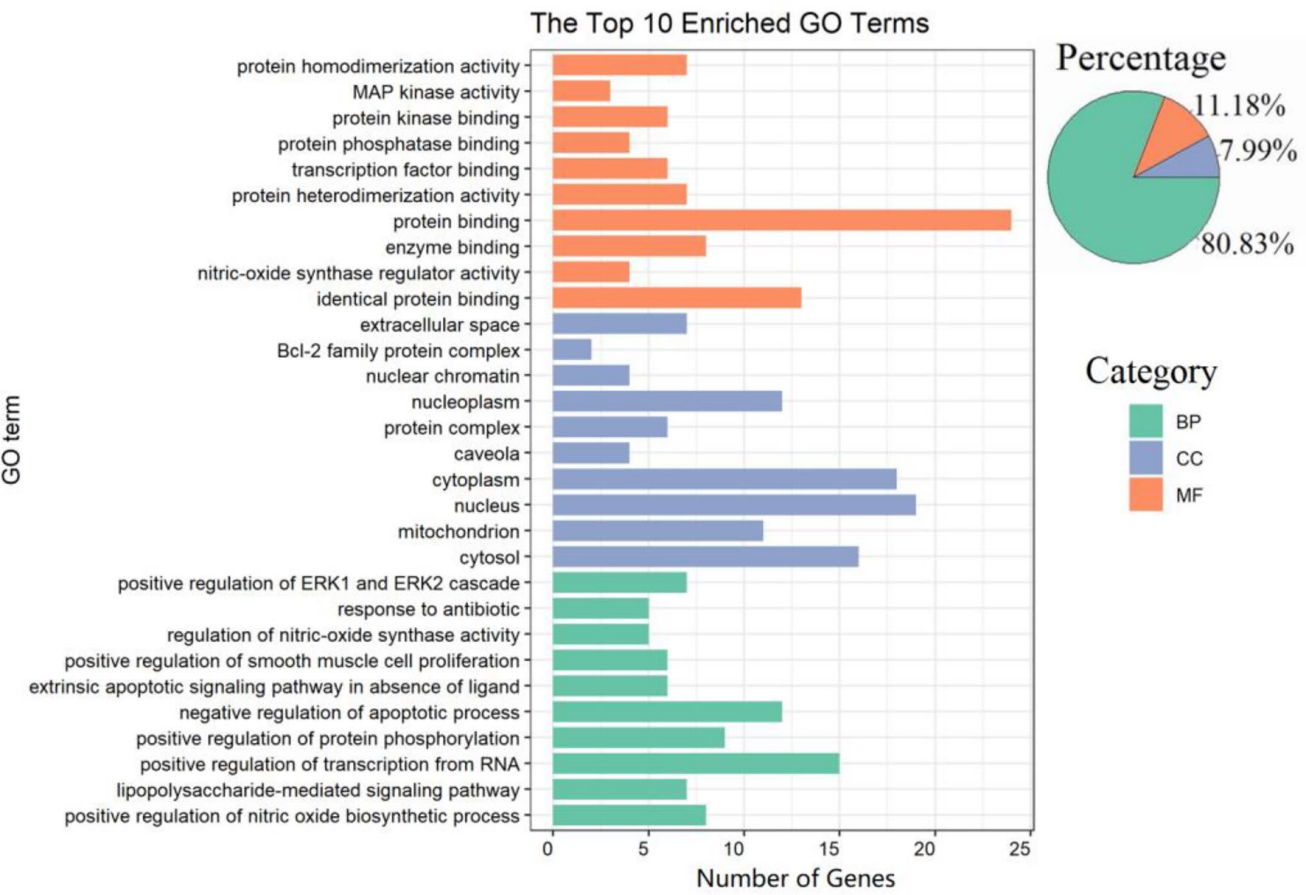
The GO functional annotation analysis. The top 10 bar chart for each category and the percentage of each category in GO term. The BP, CC and MF were represented by green, orange and gray blue, respectively [drawn by R 3.6.3 (https://www.r-project.org/)].

Through GO functional annotation analysis, 313 GO terms were obtained, including 253 biological process (BP) terms, 35 molecular function (MF) terms and 25 cellular component (CC) terms, with *p* < 0.05. Each *p*-value of enrichment results was calculated, ranking *p*-values according to the order from small to large. The pie plot showed the proportion of enriched items in each part, with BP accounting for the largest proportion at 80.83%, followed by MF and CC at 11.18% and 7.99% respectively. Different categories of BP, MF and CC were represented in green, orange and gray blue (Fig. 4). The results of GO functional enrichment analysis showed that IGEUs in the treatment of OP were mainly regulated by response to oxygen-containing compound, regulation of cell proliferation and apoptotic, response to an organic substance, regulation of protein phosphorylation in BP, and mitochondrion, nucleus, cytoplasm and nucleoplasm in CC, and protein binding, enzyme regulator activity, protein phosphatase binding and protein phosphatase binding in FM. The above analysis suggested that the active compounds of IGEUs may exert anti-OP effects by participating in various biological regulatory processes.

**Figure 5:**
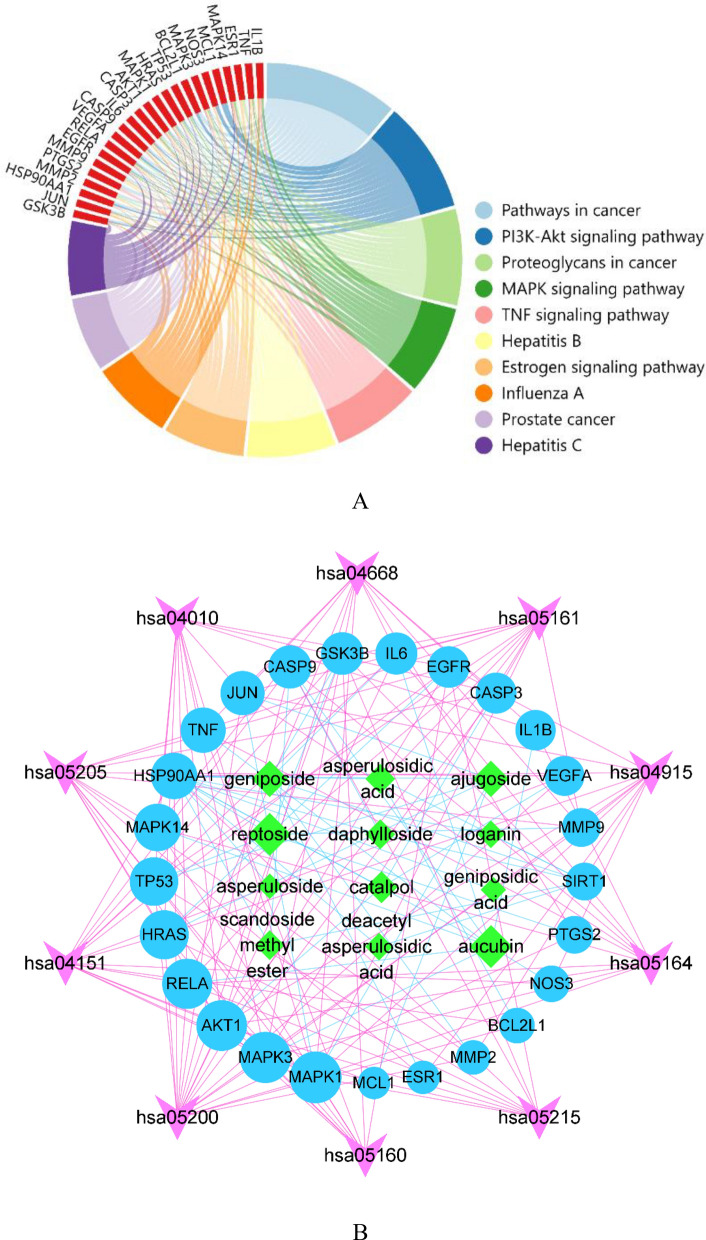
(**A**) KEGG analysis of top 10 enrichment pathways. The importance of the pathways was evaluated by *p*-value and ranked by the numbers of genes. The chord plot showed the top 10 pathway terms and corresponding targets. Different colors of the graph represented different signal pathways, and the red color was the relevant targets. The more lines in the pathway, the more targets were enriched. (**B**) The compound—hub target—pathway network. Green rhombus nodes represented the 12 active compounds. The larger the rhombus, the larger the edgecount, which means that the compound was more important. The blue circles represented the hub targets, and the purple arrow represented the KEGG pathway [created from Cytoscape 3.8.0 (https://cytoscape.org)].

The KEGG enrichment analysis revealed 105 pathway items, and the top 10 pathways were shown in Fig. 5A. The detailed information of targets and pathways were listed in Table 4. The main pathway included pathways in cancer, PI3K-Akt signaling pathway, proteoglycans in cancer, TNF signaling pathway, Hepatitis B, MAPK signaling pathway, Estrogen signaling pathway, influenza A, prostate cancer and hepatitis C. Combined with previous reports, there were three known therapeutic pathways for OP, including PI3K-Akt signaling pathway, MAPK signaling pathway and Estrogen signaling pathway^62–64^. Therefore, the above three signaling pathways and related targets were considered as the candidate pathways for further validation. The compound—hub target—pathway network, including 46 nodes and 173 edges (Fig. 5B). The main active compounds of IGEUs were distributed in different pathways and played a coordinating role in the treatment of OP. The core compounds included aucubin (edgecount = 9), reptoside (edgecount = 9), geniposide (edgecount = 6) and ajugoside (edgecount = 6).

**Table 4:** Annotation of the top 10 KEGG pathways.

ID	Description	*P*-value	Gene ID	Count
hsa05200	Pathways in cancer	1.23E−16	GSK3B, JUN, HSP90AA1, MMP2, PTGS2, MMP9, EGFR, RELA, VEGFA, CASP9, IL6, CASP3, AKT1, MAPK1, HRAS, TP53, BCL2L1, MAPK3	18
hsa04151	PI3K-Akt signaling pathway	6.12E−13	GSK3B, HSP90AA1, NOS3, EGFR, RELA, VEGFA, CASP9, IL6, AKT1, MAPK1, HRAS, TP53, MCL1, BCL2L1, MAPK3	15
hsa05205	Proteoglycans in cancer	5.23E−13	MMP2, MAPK14, ESR1, TNF, MMP9, EGFR, VEGFA, CASP3, AKT1, MAPK1, HRAS, TP53, MAPK3	13
hsa04010	MAPK signaling pathway	2.19E−10	JUN, IL1B, CASP3, MAPK1, AKT1, MAPK14, HRAS, TNF, TP53, RELA, EGFR, MAPK3	12
hsa04668	TNF signaling pathway	1.61E−14	IL6, JUN, IL1B, CASP3, MAPK1, AKT1, MAPK14, PTGS2, TNF, MMP9, RELA, MAPK3	12
hsa05161	Hepatitis B	4.92E−13	CASP9, IL6, JUN, CASP3, MAPK1, AKT1, HRAS, TNF, TP53, MMP9, RELA, MAPK3	12
hsa04915	Estrogen signaling pathway	3.98E−13	HSP90AA1, JUN, NOS3, MMP2, MAPK1, AKT1, HRAS, ESR1, MMP9, EGFR, MAPK3	11
hsa05164	Influenza A	1.20E−10	CASP9, GSK3B, IL6, JUN, IL1B, MAPK1, AKT1, MAPK14, TNF, RELA, MAPK3	11
hsa05215	Prostate cancer	6.76E−12	CASP9, GSK3B, HSP90AA1, MAPK1, AKT1, HRAS, TP53, RELA, EGFR, MAPK3	10
hsa05160	Hepatitis C	2.94E−10	GSK3B, MAPK1, AKT1, MAPK14, HRAS, TNF, TP53, RELA, EGFR, MAPK3	10

### Molecular docking

**Figure 6:**
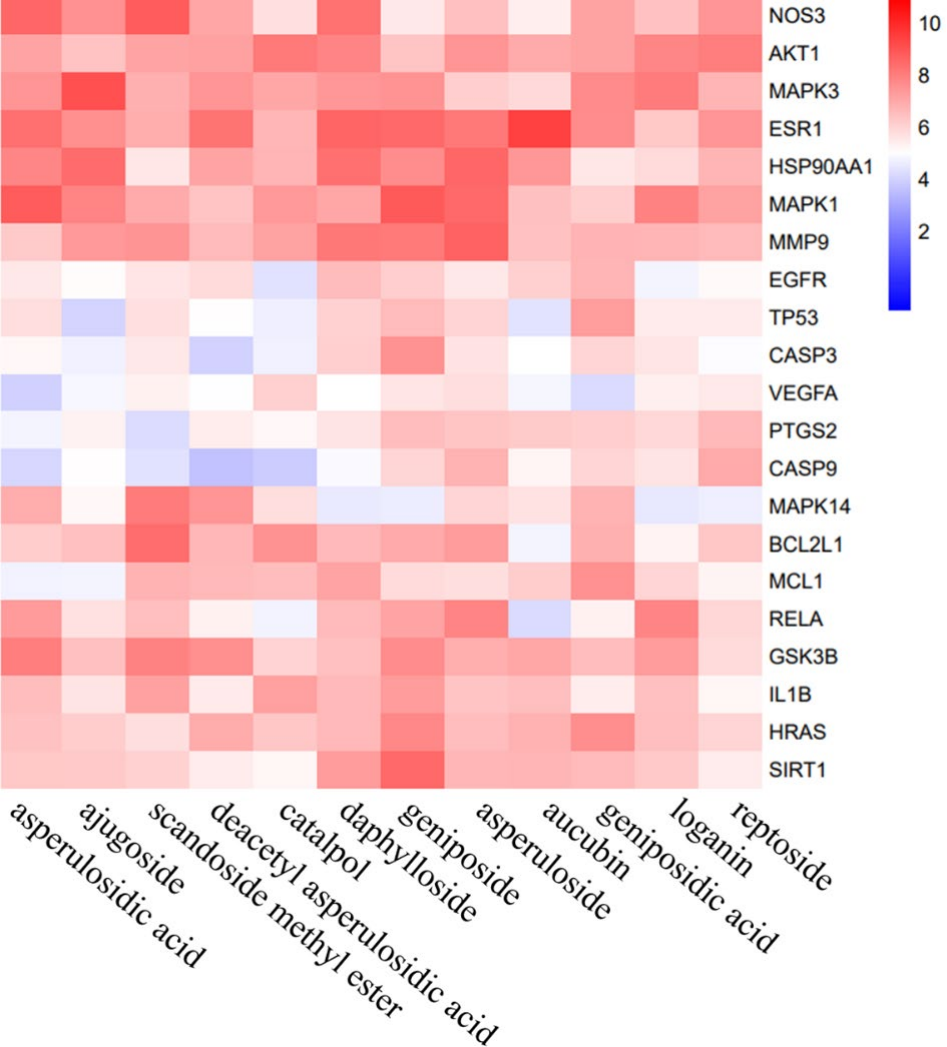
Heatmap of the molecular docking of active IGEUs with hub targets. The color represented the Total score. The redder the color, the higher the Total score, and the higher the affinity between the receptor and ligand [constructed by R 3.6.3 (https://www.r-project.org/)].

**Figure 7:**
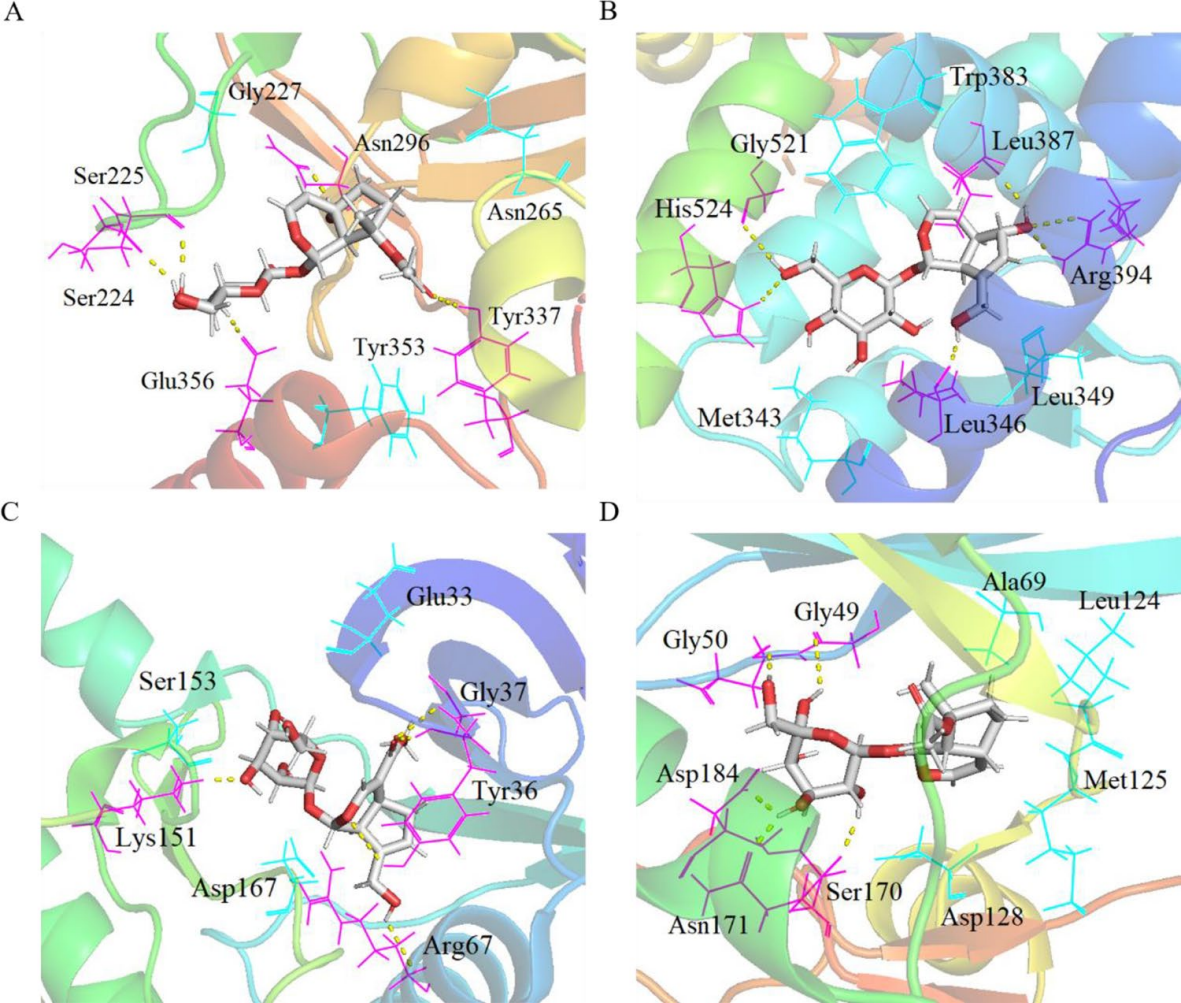
The binding model of core compounds with hub targets. The skeleton of protein was represented by bands, the active residues were represented by straight lines, the yellow dotted line represented hydrogen bonds, and the compound was shown as a sticks model. (**A**) AKT1-reptoside, (**B**) ESR1-aucubin, (**C**) MAPK1-geniposide, (**D**) MAPK3-ajugoside. [(**A**–**D**) were created from PyMOL 2.4 (https://pymol.org)].

We screened 12 IGEUs and 25 hub targets for molecular docking verification, which played a more critical role in the treatment of OP. The results showed that the Total score between most target proteins and compounds were above 4, indicating that these ligands and receptors could bind stably (Fig. 6). Moreover, we selected 4 hub targets as representative examples to show their docking modes, namely AKT1(PDB: 3OS5), ESR1(PDB ID: 6PFM), MAPK1(PDB ID: 5K4I) and MAPK3(PDB ID: 2ZOQ). The binding mode of reptoside with AKT1 showed that reptoside formed 5 hydrogen bonds with Asn296, Tyr337, Glu356, Ser224 and Ser225, formed hydrophobic interaction with the Gly227, Asn265 and Tyr353(Fig. 7A). The docking mode of aucubin and ESR1 had the highest Total score. We could observe the formation of 6 hydrogen bonds at different sites of ESR1, namely aucubin with Gly521, His524, Leu346, Arg394 and Leu387. We could also observe the hydrophobic interaction between aucubin with Trp383, Leu349 and Met343(Fig. 7B). The binding between geniposide and MAPK1 included 4 hydrogen bonds with Lys151, Arg67, Tyr36 and Gly37, and hydrophobic interaction linked with Glu33, Ser153 and Asp167(Fig. 7C). The ajugoside at the active site of MAPK3 formed 5 hydrogen bonds interactions with Gly49, Gly50, Asp184, Asn171 and Ser170. At the same time, ajugoside formed hydrophobic interactions with Ala69, Leu124, Met125 and Asp128(Fig. 7D).

### Molecular dynamic simulation and binding free energy analysis

**Figure 8:**
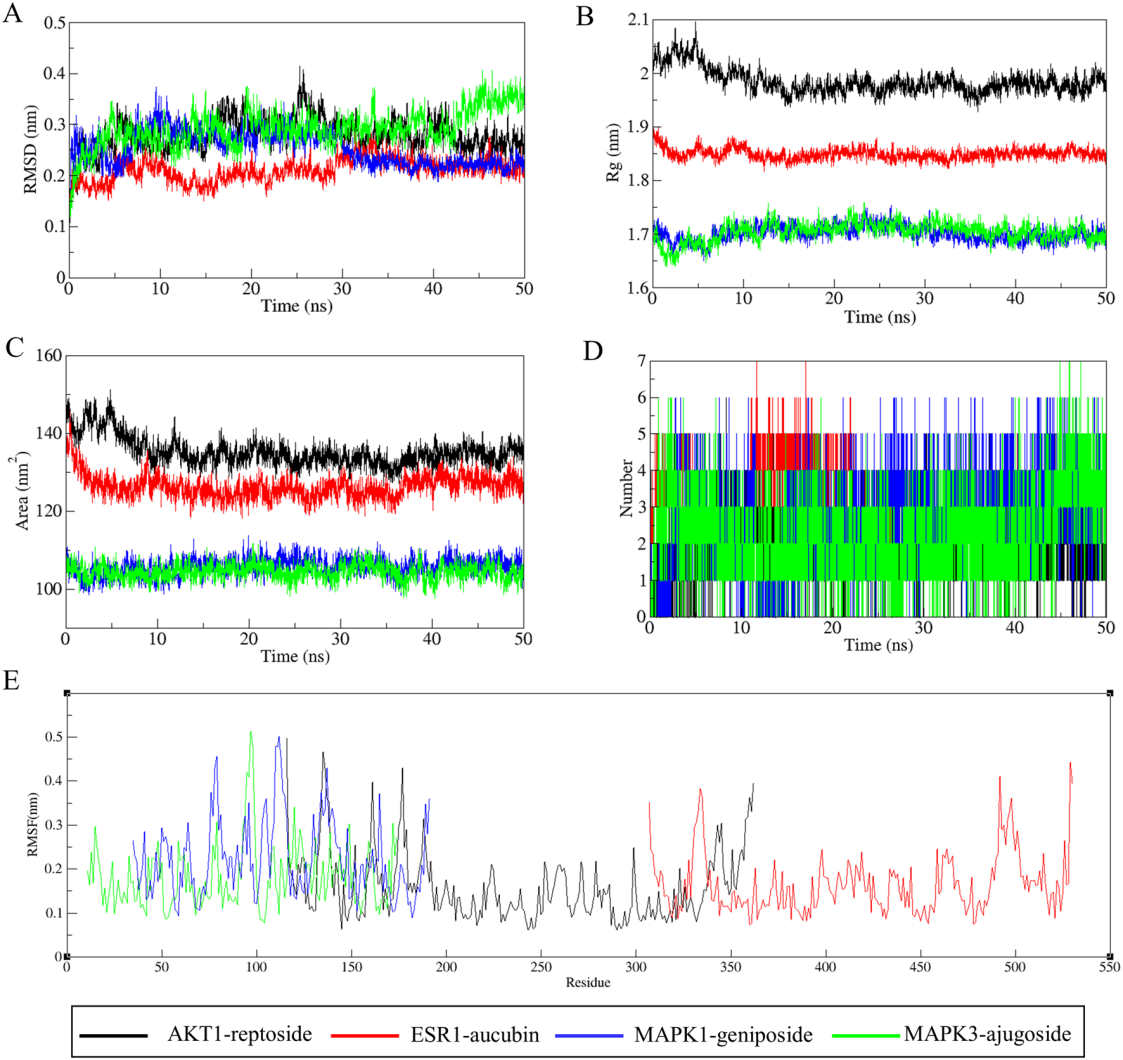
The MD simulations of the complexes of 50 ns. (**A**) RMSD: Root mean square deviations, (**B**) Rg: Radius of gyration, (**C**) SASA: solvent-accessible surface area, (**D**) Number: Number of hydrogen bonds, (**E**) RMSF: Root mean square fluctuations.

We selected the above 4 representative molecular docking complexes for further MD study. The RMSD analysis of MD trajectories showed that all systems reached equilibrium at 50 ns with minimal fluctuations (Fig. 8A). The plot of AKT1-reptoside complex showed a stable equilibrium approximately at 30 ns, then a stable RMSD could be seen approximately at ~ 0.27 nm. The ESR1-aucubin complex was stable from the beginning and had a consistent RMSD around ~ 0.2 nm. The MAPK1-geniposide complex stabilized at ~ 0.22 nm during 50 ns of MD simulation. Compared with other complexes, the MAPK3-ajugoside took a relatively slower time to reach a stable conformation. In summary, the stability of complexes was high, and the trajectories were suitable for further analysis.

In order to understand the structural stability of the protein–ligand complexes, we determined the compactness of the protein structure by computing the Rg (Fig. 8B). The Rg plots showed that the structural dynamics of AKT1-reptoside, ESR1-aucubin, MAPK1-geniposide and MAPK3-ajugoside complexes were quite stable throughout the simulation time, with mean Rg values of ~ 1.83 nm, ~ 1.64 nm, ~ 1.50 nm and ~ 2.19 nm, respectively. AKT1-reptoside complex had the highest Rg towards the whole simulation, and Rg for MAPK1-geniposide and MAPK3-ajugoside complexes were almost similar.

Moreover, the SASA of the complexes was analyzed to assess the complex volume change through the simulation trajectories (Fig. 8C). The complexes of AKT1-reptoside and ESR1-aucubin had a higher SASA profile in the initial phase, followed by a lower descriptor, and then maintained a stable SASA until the end of the simulation. The SASA of MAPK1-genpiside and MAPK3-reptoside complexes were similar, with less fluctuation during the simulation, indicating that the stability of these two complexes were less affected by the solvent.

Hydrogen bond interaction is one of the main parameters reflecting the stability of the ligand at the active site of the protein. Thus, we further investigated the changes in the numbers of hydrogen bonds (Fig. 8D). We found there was a maximum occupancy of 6 hydrogen bonds between reptoside and AKT1. Among them, 4 hydrogen bonds remained consistent until ~ 50 ns. There were up to 7 hydrogen bonds between aucubin and ESR1, which could be seen at ~ 20 ns, and 3 hydrogen bonds remained stable in the last 10 ns. MAPK1 formed up to 6 hydrogen bonds with geniposide, which were observed consistent until 50 ns. MAPK3 showed the possibility of forming up to 7 hydrogen bonds with ajugoside. The above analysis results revealed that the hydrogen bond interactions between these amino acid residues and compound were dynamic.

We further performed RMSF analysis to assess the positional fluctuation of each amino acid around its average position. The result indicated that all the complexes showed equilibrium fluctuations (Fig. 8E). The plot showed that the AKT1-reptoside and ESR1-aucubin complexes had large fluctuations at the protein terminal residues. MAPK1-geniposide and MAPK3-ajugoside complexes had similar fluctuations and showed larger fluctuations at the active sites, suggesting that greater flexibility of active residues was more favorable for ligand and protein binding.

**Figure 9:**
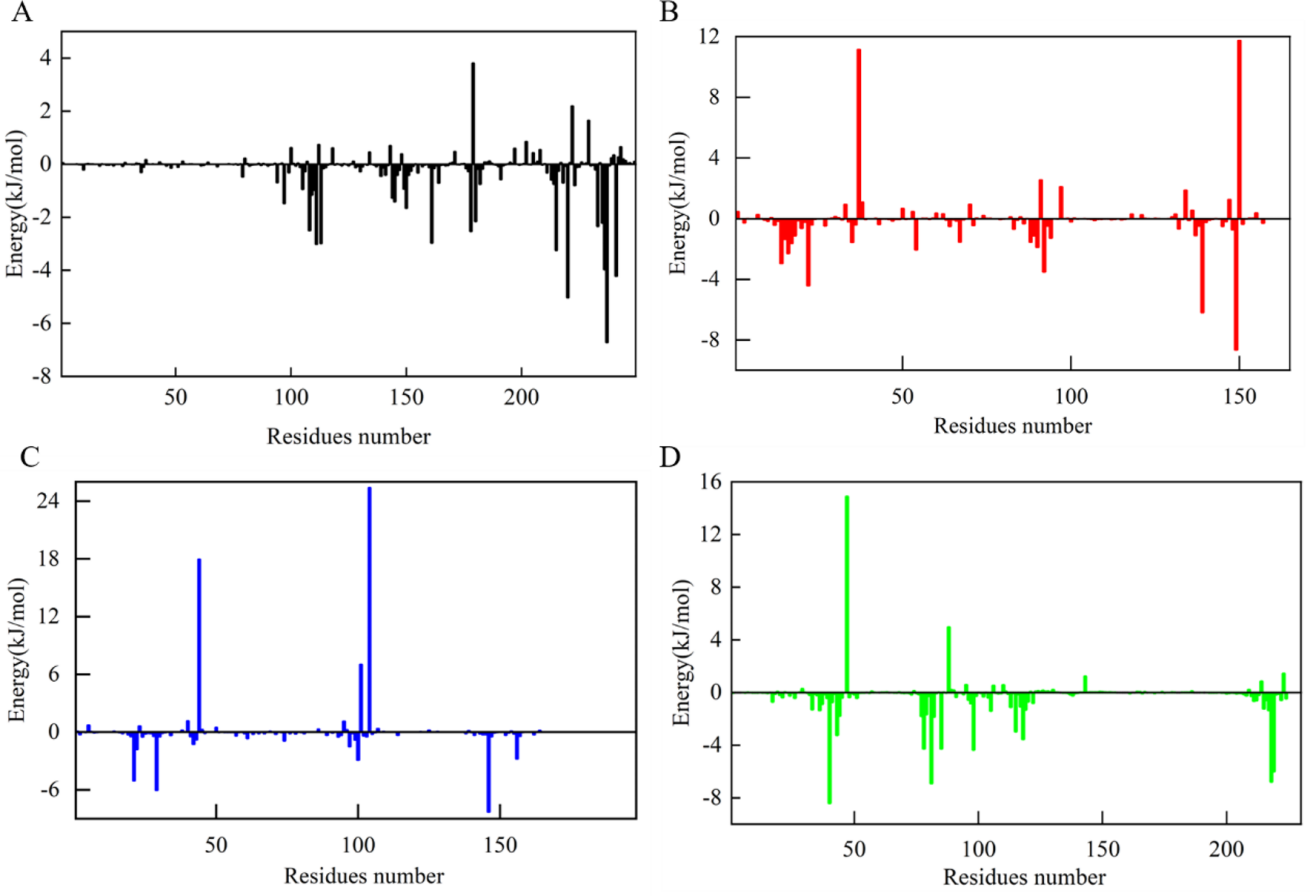
Decomposition of binding free energy for each complex. (**A**) AKT1-reptoside, (**B**) ESR1-aucubin, (**C**) MAPK1-geniposide, (**D**) MAPK3-ajugoside.

To better understand the molecular interaction and stability related to the complexes, the binding free energy was analyzed in detail. Results showed that all the binding free energy was less than zero, indicating the reaction can proceed spontaneously (Table 5). The detailed decomposition of the energy components revealed that the van der Waals energy and electrostatic interaction energies were the major contributors to the binding free energy of the complexes. The nonpolar solvation energy played a supplement role in binding. To analyze the contribution of residues to protein ligand interaction, the free energy decomposition per residue was employed (Fig. 9). Residues with energy > 5.0 kJ/mol or < − 5.0 kJ/mol were considered to be the critical residues for ligand binding to protein^65^. The calculation results showed that Trp352 and Tyr335 in AKT1 had strong interactions with reptoside (Fig. 9A). Aucubin had the lowest interaction energy with Leu346. In addition, the binding energy of aucubin with Leu387, His524 and Leu525 were also low (Fig. 9B). The binding of geniposide to MAPK1 was mainly supported by the amino acids’ residues Leu156, Val39, Ile31, Lys54 and Asp111 (Fig. 9C). Analysis of MAPK3-ajugoside complex showed that Leu346, Leu387, His524, Leu525 and Glu353 energetically favor the binding of ajugoside (Fig. 9D). Overall, the identification of critical residues in these proteins facilitated the discovery of new selective inhibitors against OP-related targets.

**Table 5:** The binding free energy of each complex and various energy components.

Complexes	ΔE_vdW_ (kJ/mol)	ΔE_elec_ (kJ/mol)	ΔG_polar_ (kJ/mol)	ΔG_SASA_ (kJ/mol)	ΔG_bind_ (kJ/mol)
AKT1-reptoside	− 158.871	− 23.789	78.68	− 16.882	− 120.861
ESR1-aucubin	− 144.328	− 110.032	184.823	− 18.807	− 88.345
MAPK1-geniposide	− 132.831	− 64.42	162.12	− 17.261	− 52.391
MAPK3-ajugoside	− 148.77	− 25.796	108.163	− 16.097	− 82.499

## Discussion

OP is becoming a major health problem with increasing age and aging bones, placing a heavy economic burden on society and families^66^. The etiology and pathogenesis of OP are still unclear. Therefore, single-target drugs cannot fundamentally prevent the development of OP^67^. TCM has the characteristics of high safety and few side effects, and it has unique advantages for complex diseases^68^. EU is the top grade of TCM, which has the effect of strengthening muscles and bones, nourishing liver and kidney^69^. Numerous studies have reported the beneficial effects of EU on skeletal and renal diseases^69,70^. But research on IGEUs is very limited. Therefore, this study was the first to explore the mechanism of IGEUs in the treatment of OP through network pharmacology and molecular simulations.

In this study, we identified effective compounds, target proteins and important pathways for IGEUs in the treatment of OP. Network analysis showed that aucubin, geniposide, reptoside and ajugoside were the core compounds of IGEUs against OP. Aucubin had strong anti-OP activity, which can not only increased the differentiation of osteoblasts, promote the increase of cortical bone thickness and bone density, but also prevented the apoptosis of osteoclasts^71,72^. Moreover, we have also reported that the total glycosides in *Eucommia ulmoides* seeds contain high content of aucubin, which could enhance bone mineral density and bone strength, suggesting that it may be a potential alternative drug for the treatment of OP^73^. Geniposide significantly promoted the formation of calcified nodules and induced osteogenic differentiation^74^. Ajugoside could resist oxidative damage in some tissues by increasing the activity of SOD^75^. Reptoside exerted good anti-inflammatory activity through inhibiting COX-1 and COX-2 enzymes^76^. Through the construction of PPI network, we identified 25 hub targets of IGEUs in the treatment of OP. These hub targets showed rich interactions with other target proteins and were also involved in 105 biological pathways. In order to find key biological pathways, we analyzed the pathways with more annotation targets and lower *p*-value. Importantly, PI3K-Akt signaling pathway, MAPK signaling pathway and Estrogen signaling pathway were related to the OP system, which were also consistent with the results reported before^62–64^. PI3K-Akt signaling pathway is an important signaling pathway for increasing osteoblast differentiation, promoting osteoclast apoptosis and inhibiting osteoclastogenesis^77^. AKT1 is a crucial signaling molecule in this signaling pathway and plays a significant role in osteogenesis^78,79^. Deficiency of AKT1 results in decreased bone mineral density throughout the body and in the femur, which is reflected in decreased femoral fracture resistance^80,81^. Many studies have shown that osteoclast generation and function can be inhibited by inhibiting the MAPK signaling pathway^82,83^. MAPKs, including p38, JNK and ERK, play an intermediary role in the regulation of bone formation. The p38, ERK1 (MAPK3) and ERK2 (MAPK1) promoted osteoblast differentiation via phosphorylation of Runx2^84^. Estrogen signaling pathway is essential for the development of OP. Estrogen is an important regulatory hormone in the human body, and its physiological role is mainly to regulate the transcription and translation of target genes by acting on the estrogen receptors of tissue cells^85^. As the major estrogen receptor subtype in bone tissue, ESR1 also plays an important role in regulating bone metabolism. Estrogen deficiency is one of the main causes of postmenopausal OP, which indicates the role of ESR1 in human bone homeostasis^86^. Studies have shown that the ratio of RANKL/OPG increased after estrogen cessation, which further leading to the increase of bone resorption^87^.

Then, molecular docking technology was used to verify the binding of hub targets with core compounds. Results showed that most hub targets had a certain binding activity, and AKT1, ESR1, MAPK1 and MAPK3 had a strong binding activity, indicating they could tightly integrate with corresponding compounds. We further study the interactions between proteins and ligands using MD simulation, which can present the fluctuation and movement of residues at any specific time in motion^88^. The RMSD showed that all complexes reached equilibrium till 50 ns, and the RMSF analysis proved the flexibility of the active amino acid residues, which facilitated the ligand binding. Similarly, the Rg and SASA analysis showed that the complexes were less affected by the solvent. Furthermore, the hydrogen bond assessment results were consistent with the results of molecular docking. The binding free energy analysis revealed that all core IGEUs showed stable binding at the binding pocket of the target proteins during the simulation time.

Through network pharmacology, molecular docking and MD simulation analysis in this study, we believed that the active compounds of IGEUs could affect the differentiation and survival of osteoclasts and osteoblast, regulate the balance of bone resorption and osteogenesis, and achieve the purpose of treating OP.

## Conclusion

The present study combined network pharmacology, molecular docking and molecular dynamic methods for the first time to reveal the pharmacological mechanism of IGEUs in the treatment of OP. Our data demonstrated that IGEUs could treat OP through a multi-component, multi-target and multi-pathway mechanism. In conclusion, this valuable finding may provide theoretical support for the clinical application of IGEUs and molecular design of therapeutic targets for OP.
